# The Mediating Effect of Negative Appearance Evaluation on the Relationship Between Eating Attitudes and Sociocultural Attitudes Toward Appearance

**DOI:** 10.3389/fpsyt.2022.776842

**Published:** 2022-03-15

**Authors:** Ruohang Wang, Youteng Gan, Xueyu Wang, Jianye Li, Małgorzata Lipowska, Bernadetta Izydorczyk, Shuai Guo, Mariusz Lipowski, Yin Yang, Hongying Fan

**Affiliations:** ^1^School of Psychology, Beijing Sport University, Beijing, China; ^2^Department of Psychology, Gdañsk University of Physical Education and Sport, Gdańsk, Poland; ^3^Institute of Psychology, University of Gdañsk, Gdańsk, Poland; ^4^Institute of Psychology, Jagiellonian University, Kraków, Poland

**Keywords:** sociocultural influence, body image, EAT-26, FNAES, SATAQ 3

## Abstract

**Objective:**

Body-image disturbance and eating disorders are significant physical and mental health problems in China. Attitudes toward the body are thought to work in conjunction with other established risk factors for dietary pathology, which include body dissatisfaction, dieting, and negative effects. Negative appearance evaluation may be valuable for extending our understanding of measuring factors and potential causal relationships associated with body image and eating problems. Therefore, this study aimed to determine the association between negative appearance evaluation and a combination of eating attitudes and sociocultural attitudes toward appearance and the mediating effect of negative appearance evaluation on the relationship between eating attitudes and sociocultural attitudes toward appearance.

**Methods:**

We invited 339 Chinese adults to undergo the Eating Attitudes Test (EAT-26), the Fear of Negative Appearance Evaluation Scale (FNAES), and the Sociocultural Attitudes toward Appearance (SATAQ-3) scale, and used AMOS 24.0 for model construction and mediating effects testing.

**Results:**

There was a significant positive correlation between EAT-26 scores and FNAES (*p* < 0.001) and SATAQ-3 scores (*p* < 0.001), and all dimensions except internalization general were significantly positively correlated (*p* < 0.05). There was a significant positive correlation between FNAES and SATAQ-3 scores for all dimensions (*p* < 0. 01). There was no significant direct effect of eating attitude on sociocultural attitude toward appearance; however, there was a significant mediating effect of fear of negative appearance evaluation.

**Conclusion:**

Our results demonstrate that negative appearance evaluation fully mediates the relationship between eating attitudes and sociocultural attitudes toward appearance. An individual’s attitude toward eating affects negative body perceptions and thus their perception of their own body shape. Our exploration of the specific effects of eating attitudes on body perception provides a psychological basis for guidance and developing interventions regarding attitudes toward the body.

## Introduction

An eating disorder is a type of mental disorder that is characterized by abnormal eating habits or a group of abnormal eating behaviors and negatively impacts physical and mental health (1, 2). Abnormal eating behaviors include reduced physical fitness, altered body weight and body fat percentage, and poor bone health (3). Moreover, eating disorders are a major cause of physical and mental health problems among young women (4). The main clinical manifestations are bulimia nervosa, anorexia nervosa, and binge-eating disorder, and the main subclinical manifestations are diet and bulimia (5, 6). Clinical eating disorders are relatively rare and primarily develop and evolve from subclinical eating disorders, which are highly common. Subclinical eating disorders affect 60% of girls and 30% of boys (7).

It has been shown that when individuals are faced with stressful events, have long-term distorted cognition of physique or body shape, or are affected by negative emotions, they are prone to abnormal eating attitudes, which often manifest as overeating, dieting, and other eating problems (8–10). Eating attitudes are a series of cognitive, emotional, and behavioral tendencies of individuals who consider eating as an object. In severe cases, these tendencies can develop into a clinical eating disorder, such as bulimia nervosa, anorexia nervosa, and binge-eating disorder (11). The Eating Attitudes Test (EAT-26) is a reliable and valid instrument that provides an objective measure of the symptoms of anorexia nervosa (12). Furthermore, the EAT-26 may be considered the most appropriate tool as an outcome measure for clinical groups or as a screening tool for high-risk groups for anorexia nervosa in non-clinical settings (13). It is influenced by external stimuli, situations, and the individuals themselves (14).

Individuals with eating disorders are prone to negative self-evaluation, which results in a deformed body image (15, 16). The most prominent psychological problems in individuals with eating disorders are related to their attitudes toward thinness and food. The heavier their weight, the stronger their desire to be thin, and the greater the difficultly in controlling excessive eating. However, underlying these specific attitudes toward body shape and eating, patients with eating disorders may also have other psychological problems, such as pursuing perfection and fear of growth and maturity (17, 18). According to Garner et al. (19), individuals with abnormal eating attitudes may have distortions in body image and inner feelings, which are accompanied by psychophysiological symptoms, such as negative body self, unreasonable eating, and irregular sleep. Previous research has shown that the total score of eating is positively correlated with the fat subscale score of the Negative Physical Self Scale, which indicates that individuals with a high eating attitude score are more likely to have an eating disorder and a more negative cognition of their body (20). Chen (21) showed that anorexia nervosa patients have negative cognitive eating disorders and body image disorders; they are not satisfied with their current body mass and may even have a cognitive bias toward their already low body mass index (BMI). However, patients with anorexia nervosa may overestimate their body weight due to visual distortions (22). Moreover, based on stereotypes about weight, people with eating disorders have double standards that lead to a distorted evaluation or characterization of their external image (23). Furthermore, personality types have been shown to influence individuals’ perceptions of body satisfaction and body image (24). There is also evidence that overvaluation of low body weight and fear of weight gain cannot be explained by general cognition, rather, they require individual judgments (26). In other words, patients do not exhibit changes in physical representations in general; instead, they present with top-down cognitive-emotional distortions in evaluations of their own bodies (27).

In 1962, Bruch first proposed that anorexia nervosa patients have body-image disorder, and numerous reports have since reported similar findings. Body-image disturbances are usually associated with eating disorders (28). Body image disorder is a distorted understanding of one’s image, which is caused by social, psychological, or biological factors (29). A disturbance in body image is an individual’s cognitive maladjustment of their body, which usually leads to negative emotional experiences (30). Thompson et al. (31) proposed the definition, “a persistent report of dissatisfaction, concern, and distress that is related to an aspect of appearance… [and] some degree of impairment in social relations, social activities, or occupational functioning” (p. 11). Body-image disturbances are particularly prominent among female college students and include dissatisfaction with body shape, skin color, height, and other aspects (32), which leads to low self-esteem, anxiety, and depression due to public self-consciousness (33). The Fear of Negative Appearance Evaluation Scale [FNAES; (34)] is used to assess apprehension about appearance evaluation and determines the degree to which people experience apprehension at the prospect of being evaluated negatively for their appearance. It was developed by modifying items from the Brief Fear of Negative Evaluation Scale (35) and creating novel items that index apprehension toward a negative appearance evaluative experience.

Thomas et al. (34) and Leary (35) speculated that fear of negative appearance evaluation (FNAE) and fear of negative evaluation (FNE) are related to sociocultural factors. Various disparate influences have received attention in the field of body image disturbances and eating disorders, including interpersonal and sociocultural factors such as negative appearance-related feedback (teasing), modeling of dieting and body image concerns by parents and peers, elevated tendencies to compare one’s appearance to others, sexual abuse, sexual harassment, and internalization of media images and messages (36, 37). Furthermore, investigating the more specific FNAE may be valuable for extending recent work regarding the identification of factors that are associated with, and potentially the cause of, body image and eating problems (51).

Appearance-ideal internalization and appearance pressures are empirically supported risk factors for body image disturbance and disordered eating in Western countries (39, 40). The sociocultural theory has been shown to be a useful framework for exploring how environmental influences contribute to body image concerns (31). The Sociocultural Attitudes toward Appearance Questionnaire (SATAQ-3) is used to document an individual’s recognition of their awareness of a societal influence, alongside the endorsement/acceptance of the prevailing message of an internalization of the touted standard (41). Research findings consistently support the strong relationship between sociocultural influences and body dissatisfaction (42). Several studies have shown that thin-ideal internalization and perceived appearance pressures are uniquely associated with and predict body dissatisfaction. Girard et al. (43) emphasized the usefulness of sociocultural models in understanding women’s body image, which includes their drive for thinness and muscularity concerns. As Knauss et al. (44) expected, internalization, pressure, and BMI contribute to the prediction of body dissatisfaction in boys and girls; moreover, girls internalize media body ideals to a greater extent and feel more pressure from media to conform to this ideal than do boys. In the model by Lovering et al. (45), sociocultural pressure from the media, partners, and peer variables significantly predict body image and eating concerns through the mediating effect of the latent thin-ideal variable (i.e., internalization of the thin-ideal and appearance comparisons), and of the four pathways, the pathway between the media influence variable to the thin-ideal variable was the strongest (45). Exploring the Tripartite Influence Model of body dissatisfaction in postpartum women. There is strong evidence for significant relationships among the putative risk factors, mediators (internalization, comparisons, and peer suppression of feelings), and criterion variables (body dissatisfaction, drive for thinness, bulimic symptoms, and self-esteem) (46). Peer influences, body-image dissatisfaction, eating dysfunction, and self-esteem in adolescent girls. Appearance-ideal internalization refers to the acceptance of culturally endorsed appearance ideals (e.g., thinness for females and muscularity for males) as one’s personal appearance standard (47), whereas perceived pressures can be defined as the feeling of being encouraged to modify one’s physical appearance to reach such ideals (48). A meta-analysis revealed that the emphasis of mass media on the beauty of being slim is significantly related to body image disorder in college students (especially women) (49). In addition, other studies have found that the likelihood of females developing an eating disorder is positively correlated with the degree of recognition of social values (50).

In summary, this study hypothesized that there are two stages of the effect of dietary attitudes on sociocultural attitudes toward appearance. In the first stage, a disorder of dietary attitudes activates FNAE. In the second stage, FNAE eventually affects sociocultural attitudes toward appearance. Therefore, this study hypothesized that: (1) there is a relationship between negative appearance evaluation and sociocultural attitudes toward appearance, and (2) FNAE plays a complete mediating role in the influence of eating attitudes on sociocultural attitudes toward appearance.

## Materials and Methods

### Participants and Procedure

Participants were 339 healthy people (176 female, 163 male) from various regions of China who were invited to complete the questionnaires. The average age of participants was 31.06 years (range = 18–72 years, standard deviation [SD] = 11.87 years), and mean BMI was 22.24 (range = 14.5–37.2, SD = 3.86). Inclusion criteria were: aged ≥18 years, Chinese nationality, residence in China, and no physical disability or somatic diseases that prevent physical activity. Criteria were verified according to responses to questions on sociodemographic data and health, which allowed for the determination of the exclusion factors.

During the data collection period, 632 participants completed the survey in Chinese. We excluded 201 returned questionnaires from the analysis because of errors in completing the questionnaires (incomplete data) and respondents who had a nationality other than Chinese. A further 92 questionnaires were excluded because the inclusion criteria were not fulfilled.

The procedure carried out in this study consisted of an online survey conducted as part of an international research project registered on ClinicalTrials.gov.^1^ This study used data collected from a Chinese population between July 20, 2020, and November 24, 2020, during the period of the global pandemic situation. The work was carried out according to the Code of Ethics of the World Medical Association (Declaration of Helsinki) for experiments involving humans. The protocol was approved by the Ethics Board for Research Projects at the Institute of Psychology, University of Gdańsk, Poland (decision no. 33/2020). The questionnaires that formed this study took around 30 min to complete. All survey participants provided electronic informed consent before registration.

### Instruments

#### Fear of Negative Appearance Evaluation Scale

The FNAES (51) was used to assess women’s apprehension toward appearance evaluations. The FNAES contained six items (e.g., I’m worried about what people think of my appearance), which were scored on a five-point Likert scale, from definitely disagree to definitely agree. Higher scores indicated greater FNAE. Cronbach’s alpha for the FNAES in the present study was 0.93.

#### The Eating Attitudes Test

The EAT-26 was used to assess subjects on their cognitive, emotional, and behavioral tendencies regarding diet (19). It comprised 26 items that measured three factors: dieting, bulimia, and food preoccupation (abbreviated as bulimia), and oral control. In this study, scale scores were calculated using screening scores. Questions 1 to 25 were scored as never/rarely/sometimes = 0, often = 1, usually = 2, or always = 3, and question 26 was scored in the opposite way. Higher scores represent more severe eating attitudes and behavioral problems. A total score ≥20 indicated the possible presence of an eating disorder. Cronbach’s alpha for the EAT-26 in the present study was 0.72.

#### The Sociocultural Attitudes Toward Appearance Questionnaire-3

The SATAQ-3 (48) was used to assess participants’ sociocultural attitudes toward appearance. The SATAQ-3 is a 30-item measure comprising four subscales: internalization general, internalization athlete, perception of pressure, and information. Each item was scored on a five-point Likert scale, from definitely disagree to definitely agree.

The information subscale consisted of nine items, which were used to measure recognition of the social importance of beauty ideals in the media (e.g., magazine advertisements are an important source of information about fashion and “being attractive”). The pressure subscale consisted of seven items, which were used to measure perceived stress to meet the Western ideal displayed by the media (e.g., I’ve felt pressure from television or magazines to have a perfect body). The internalization general subscale consisted of nine items, which were used to measure the internalization of the thin ideal (e.g., I compare my body to the bodies of people who are on television, and I do not care if my body looks like the body of people who are on television). The internalization athlete subscale consisted of five items, which were used to measure the internalization of the sports ideal (e.g., I try to look like sports athletes and I wish I looked as athletic as sports stars). Previous studies have shown that the SATAQ-3 has high internal consistency in female college students and patients with eating disorders. Cronbach’s alpha for the SATAQ-3 in the present study was 0.88.

### Data Analysis

Data analysis was performed using SPSS 26.0 and AMOS 24.0. *T*-tests or one-way analyses of variance (ANOVAs) were used to compare scores between participants with different demographic information. Pearson correlation analysis was used to explore relationships between FNAES, EAT-26 (dieting, bulimia and food preoccupation, and oral control), and SATAQ-3 (information, pressure, internalization general, and internalization athlete) scores. In addition, AMOS 24.0 was used to clarify the influencing path of participants’ eating attitudes and sociocultural attitudes toward appearance on external apprehension toward appearance evaluations. Bootstrapping was used to test the mediating effect of apprehension toward appearance evaluations on the relationship between eating attitudes and external sociocultural attitudes toward appearance. A *p* < 0.05 was considered statistically significant.

## Results

Table 1 shows the differences between the selected demographic variable groups for the FNAES, EAT-26, and SATAQ-3 scores. The mean FNAES score was higher in females (17.37 ± 6.19) than in males (16.41 ± 5.67). Females scored lower than men on the EAT-26 for both total and all dimension scores. SATAQ-3 scores showed similar patterns as the FNAES and EAT-26 scores; however, there were no significant differences.

**TABLE 1:** Participants’ scores on the FNAES, EAT-26, and SATAQ scales.

Variables	Category (N)	FNAES	EAT-26				SATAQ			
			Dieting	Bulimia	Oral control	Total score	Internalization general	Internalization athlete	Pressure	Information
Gender	Male (176)	16.41 ± 5.67	6.49 ± 4.90	0.97 ± 1.78	2.14 ± 2.46	9.60 ± 6.54	25.93 ± 7.08	15.17 ± 2.47	20.68 ± 7.03	26.72 ± 5.14
	Female (163)	17.37 ± 6.19	4.94 ± 4.69	0.82 ± 1.31	1.60 ± 2.06	7.36 ± 5.99	24.55 ± 6.61	15.02 ± 2.84	18.99 ± 6.74	25.23 ± 4.88
	*T*-value	−1.50	2.97	0.87	2.16	3.27	1.85	0.51	2.25	2.72
	Cohen’s d	0.16	0.32	0.10	0.24	0.36	0.20	0.06	0.25	0.30
Age	≤24 years (140)	18.37 ± 5.65*^a^*	5.04 ± 5.28*^a^*	1.02 ± 1.73	2.29 ± 2.45*^a^*	8.35 ± 6.87*^a^*	25.44 ± 6.71	15.28 ± 2.86	20.51 ± 6.55	25.4 ± 5.13*^a^*
	25∼34 years (109)	17.06 ± 6.10*^ac^*	6.99 ± 4.67*^bc^*	1.05 ± 1.57	2.19 ± 2.32*^bc^*	10.23 ± 6.55*^bc^*	25.13 ± 6.25	15.34 ± 2.45	20.04 ± 6.87	27.54 ± 4.73*^bc^*
	35∼44 years (42)	14.29 ± 5.36*^b^*	5.86 ± 4.74*^ac^*	0.52 ± 1.25	0.60 ± 1.17*^a^*	6.98 ± 5.15*^a^*	24.14 ± 7.64	14.14 ± 2.23	18.02 ± 7.81	24.21 ± 5.30*^a^*
	45∼54 years (26)	14.77 ± 4.89*^bc^*	4.73 ± 3.63*^a^*	0.65 ± 1.44	1.65 ± 2.21*^ac^*	7.04 ± 4.74*^a^*	25.23 ± 7.97	14.65 ± 3.25	19.92 ± 7.93	26.19 ± 4.87*^ac^*
	≥55 years (22)	13.82 ± 5.95*^b^*	5.05 ± 3.20*^ac^*	0.41 ± 1.05	0.41 ± 0.91*^b^*	5.86 ± 3.34*^a^*	27.05 ± 8.27	15.14 ± 1.81	18.45 ± 6.46	25.41 ± 4.31*^ac^*
	F value	7.00***	3.02*	1.75	7.90***	4.05**	0.68	1.96	1.29	4.59**
	η^2^	0.08	0.04	0.02	0.09	0.05	0.01	0.02	0.02	0.05
BMI	≤18.4 Thin (40)	17.73 ± 5.44*^a^*	4.33 ± 4.97	1.53 ± 2.48*^a^*	3.90 ± 3.26*^a^*	9.75 ± 7.20	24.30 ± 5.64	15.23 ± 3.31*^a^*	22.00 ± 6.86*^a^*	25.93 ± 4.40
	18.5∼23.9 Normal (206)	17.39 ± 6.04*^ac^*	6.05 ± 5.03	0.92 ± 1.38*^bc^*	1.97 ± 2.11*^bc^*	8.94 ± 6.45	25.90 ± 6.72	15.41 ± 2.56*^ac^*	20.65 ± 6.49*^ac^*	26.55 ± 5.04
	24.0∼27.9 Overweight (63)	15.65 ± 6.23*^a^*	5.75 ± 4.70	0.57 ± 1.42*^bc^*	0.78 ± 1.44*^b^*	7.10 ± 6.16	24.32 ± 7.48	14.52 ± 2.49*^a^*	17.32 ± 7.48*^b^*	25.02 ± 5.28
	≥28 Obesity (30)	14.77 ± 4.31*^b^*	5.53 ± 3.42	0.60 ± 1.38*^bc^*	0.90 ± 1.24*^b^*	7.03 ± 4.26	24.2 ± 8.00	14.03 ± 2.24*^a^*	17.03 ± 6.88*^b^*	24.43 ± 5.16
	F value	3.00*	1.43	3.47*	20.13***	2.42	1.50	3.65*	7.00***	2.60
	η^2^	0.03	0.01	0.03	0.15	0.02	0.01	0.03	0.06	0.02

*p < 0.05, **p < 0.01, ***p < 0.001; ps valued smaller than 0.05 were bolded for clarity. And one-way analysis of variance tests (ANOVA) was used for analyses by age, residence, or BMI. abc the same letter means that there is no difference between pairwise comparisons within the group and different letters mean that there is a difference. (N = 339, Mean ± SD).

Pearson correlation analysis showed that FNAES score was positively correlated with the dieting (*r* = 0.18, *p* < 0.01), bulimia (*r* = 0.18, *p* < 0.01), and total score (*r* = 0.19, *p* < 0.05) of the EAT-26, and internalization general (*r* = 0.30, *p* < 0.01), internalization athlete (*r* = 0.26, *p* < 0.01), pressures (*r* = 0.37, *p* < 0.01), and information (*r* = 0.31, *p* < 0.01) of the SATAQ-3. In addition, the bulimia score was positively correlated with the pressures (*r* = 0.11, *p* < 0.05) and information scores (*r* = 0.13, *p* < 0.05), the oral control score was positively correlated with the information score (*r* = 0.14, *p* < 0.05), and the total score was positively correlated with the information score (*r* = 0.16, *p* < 0.01) (Table 2).

**TABLE 2:** Correlation between FNAES, EAT-26, and SATAQ-3 scores.

	FNAES	Dieting	Bulimia	Oral control	EAT-26 (total score)	Internalization general	Internalization athlete	Pressure	Information
FNAES	1								
Dieting	0.18**	1							
Bulimia	0.18**	0.37**	1						
Oral control	0.04	0.08	0.27**	1					
EAT-26 (Total score)	0.19**	0.88**	0.63**	0.48**	1				
Internalization general	0.30**	−0.04	0.08	−0.06	−0.03	1			
Internalization athlete	0.26**	−0.01	−0.01	0.01	−0.01	0.36**	1		
Pressure	0.37**	−0.05	0.11*	0.08	0.02	0.68**	0.46**	1	
Information	0.31**	0.11	0.13*	0.14*	0.16**	0.55**	0.32**	0.54**	1

*p < 0.05, **p < 0.01; ps valued smaller than 0.05 were bolded for clarity.

**FIGURE 1:**
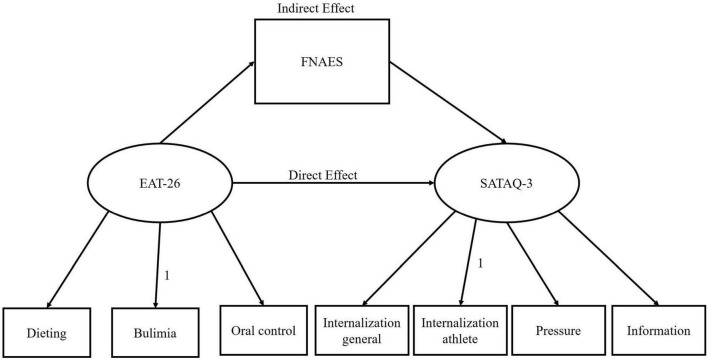
Path analysis of the relationship between FNAES, EAT-26, and SATAQ-3 scores of the participants.

The structural equation models built using the EAT-26 score as the independent variable, SATAQ-3 score as the dependent variable, and FNAES score as the mediating variable (Figure 1) showed that the model had a good fit (χ^2^/df = 2.831, AGFI = 0.931, CFI = 0.945, GFI = 0.966, IFI = 0.946, RMSEA = 0.074, SRMR = 0.045, TLI = 0.915). Furthermore, the standardized path coefficients showed that the EAT-26 score significantly predicted the FNAES score (β = 0.21, *p* < 0.01), and the FNAES score significantly predicted the SATAQ-3 score (β = 0.41, *p* < 0.01) (Figure 1).

The results of the mediation effect indicated that the mediating effect was significant; however, the direct effect was not significant. Apprehension toward appearance evaluations played a complete mediating role between eating attitudes and sociocultural attitudes toward appearance, and the mediation effect accounted for 70.09% of the total effect (Table 3).

**TABLE 3:** Bootstrap analysis of the mediation effect size and significance test of FNAES in EAT-26 and SATAQ.

Path	Standardized effect size	Standard error	Effect size	p	95%CI
	(Effect)	(Boot SE)	(%)		
EAT-26→SATAQ	0.04	0.12	29.91	0.40	[−0.19, 0.19]
(Direct effect)					
EAT-26→FNAES→SATAQ	0.08**	0.07	70.09	0.01	[0.01, 0.29]
(Mediation effect)					
EAT-26→SATAQ	0.12*	0.09	100.00	0.04	[0.01, 0.38]
(Total effect)					

*p < 0.05, **p < 0.01; ps valued smaller than 0.05 were bolded for clarity.

## Discussion

The present study examined the association between negative appearance evaluation and a combination of eating attitudes and sociocultural attitudes toward appearance. We also explored the mediating effect of negative appearance evaluation on the relationship between eating attitudes and sociocultural attitudes toward appearance. Overall, the data suggested that negative appearance evaluation is associated with sociocultural attitudes toward appearance. Specifically, those who report eating disorders demonstrated higher levels of sociocultural attitudes toward appearance. Negative appearance evaluation scores significantly mediated the relationship between eating attitudes and sociocultural attitudes toward appearance.

These findings both support and extend previous literature regarding the relationship between sociocultural attitudes toward appearance and adaptive eating patterns. Women are at higher risk of eating disorders, especially women in college, and have the highest prevalence of eating disordered behaviors (40, 52). The sex difference might be attributed to biological, psychodynamic, and sociocultural factors (53). However, there was no significant difference in eating attitudes between different BMI groups, except for bulimia and oral control subscale scores. Furthermore, those aged 25–34 years had the highest average score on this scale. Current research on eating disorders is focused on adolescents (7, 54–56), and there is a lack of large-scale cross-sectional studies on the age of onset of eating disorders, which may be a useful research direction in the future. This study showed that there was no significant difference in negative image ratings between men and women. However, there were significant differences in the evaluation of one’s own image among different age groups; the perception of one’s appearance and physique became more negative with age. As women approach midlife, the pressures that came from body monitoring and surveillance (57) begin prior to middle age. These pressures may become more salient during the aging process (58, 59). In addition to the social ideal of being beautiful and slim in women of all ages, middle-aged women also face age-related pressures, such as the need to avoid wrinkles and other visible signs of aging (60–62). These increasing pressures may develop into concerns about appearance, which increases the fear of negative appearance judgment and results in greater dissatisfaction with their body (63). Scores differed significantly between BMI groups, whereby people with a lower BMI tended to have a more negative evaluation of their appearance. This was not consistent with the results of previous studies. Studies have found that the FNAES does not significantly correlate with BMI (51). This discrepancy may be due to cultural differences between China and the West; moreover, the pursuit of thinness for a prolonged period may lead people with a lower BMI to be less satisfied with their appearance. Notably, the impact of the coronavirus 19 (COVID-19) outbreak is a problem that cannot be ignored. The COVID-19 outbreak threatened public physical health while having a profound impact on the public’s mental health (64, 65). Indeed, several self-reported studies have suggested that dietary restrictions or compensatory behaviors were further exacerbated during the outbreak (66). Moreover, recent questionnaire data suggests that the epidemic had a similar negative impact on people’s diet under certain conditions, whereby symptoms became more severe in patients with eating disorders (67). In addition, under the strict control conditions of the COVID-19 pandemic in China, such effects may have been amplified, which likely affected individuals’ perception of their external image. To some extent, this may have influenced a more negative view of their external image, which can lead to cognitive biases that are ultimately reflected in sociocultural attitudes.

This study also found a positive correlation between eating attitudes and FNAE, which is consistent with previous research. Several studies have investigated eating disorders in both men and women and found that those with eating disorders have a higher incidence of emotional disorders (68–73). Furthermore, satisfaction with body image is significantly different from that of the control group (53), demonstrating clear body dissatisfaction in this group (74, 75). FNAE, which is defined as social anxiety and distress due to their appearance negatively evaluated, has been found to be predictive of eating disorders above and beyond other body image variables (51). Patients with eating disorders may be more worried about their appearance being negatively evaluated. Indeed, clinical samples of women with eating disorders report higher levels of fear of negative evaluation than that of controls (76). Social appearance anxiety (i.e., fear of negative evaluation of one’s appearance) and general fear of negative evaluation have each been proposed as risk factors for eating disorders. It is possible that treating FNAE may reduce eating disorder symptoms (77).

Fear of negative appearance evaluation scale was also associated with sociocultural attitudes toward appearance, and we observed a correlation between eating attitudes and information. Because of the cultural differences between the West and China, people have different sociocultural attitudes toward appearance, which affects the generalization of our results. Slimness is an ideal criterion for female attractiveness in China (78), and girls who do not conform to this philosophy are more likely to receive negative feedback, experience physical dissatisfaction, and perform inappropriate compensatory behaviors to reduce the difference between their own and the ideal body shape (78). Moreover, there is a stronger cultural premium on appearance and attractiveness as defining attributes of femininity in China (79). In Western culture, objectification theory (80) suggests that Western societies sexually objectify the female body, which leads women to feel as if they are constantly being valued (or devalued) according to their appearance. Internalization of media images and messages (36, 81) may lead to the onset and maintenance of body image disturbance and eating disorders (47, 82). The external pressure and information that constantly instill the concept of beauty may influence eating attitudes toward gaining social recognition of “beauty” through dieting and other behaviors.

Our findings contribute to evidence suggesting that the relationship between eating attitudes and sociocultural attitudes toward appearance is mediated by negative appearance evaluation. van den Berg et al. (83) reported that girls exhibit internalization of the ideal body and feel more pressure from the media than boys. When girls have a strong negative image of their bodies, there is a greater internalizing effect when they see “plus-size” models or “slim” pictures that suggest that slim models represent beauty, and a negative self-image reinforces this perception. The age range of participants in this study was largely focused on adolescence and the period of transition from adolescence to middle age. Body image concerns usually begin and increase during early adolescence and decrease in younger adulthood; thus, it affects mental health at different stages of life. The type and extent of body image disturbance vary with age, race, peers, family, and sociocultural influences. The development of negative body image disorders is considered to be the strongest determinant of sociocultural influences, and body dissatisfaction is considered to be a predictor of eating disorders because adolescents do not accept the “ideal body.” Furthermore, current research suggests that body dissatisfaction is associated with an increased tendency to initiate unhealthy eating behaviors, for which girls are at higher risk than boys (84). Notably, the present study was conducted in adolescents without a diagnosis of eating disorders in a non-clinical setting. In an earlier study, Furnham et al. (85) observed that males and females had comparable levels of body dissatisfaction, but in opposite directions: 41% of males wanted to be thinner, whereas 22% of females wanted to gain weight. Thus, underweight men seem to be dissatisfied with their bodies, whereas underweight women seem to be very satisfied (86). Although both men and women desire physical perfection, gaining weight and attaining the ideal body should be achieved through exercise and weight training and not only *via* diet control (87). However, the idea of achieving the socio-cultural perception of a perfect body, combined with girls’ dissatisfaction with their bodies, can exacerbate inappropriate eating behaviors and increase their risk of developing eating disorders (88). Many individuals with eating disorders are undiagnosed (89) and thus do not receive the necessary support and training. Adolescence is a time when individuals are highly vulnerable psychologically; therefore, it is important to provide the necessary support to enable them to develop into healthy adults. Adolescents require education around perceiving their bodies in a healthy way and how to filter messages in the mass media to prevent negative effects. In a previous study, Posavac et al. (90) observed that female students with a negative self-image who received psycho-educational instruction in media analysis were less likely to make social comparisons and less vulnerable to the negative effects of “thin” images of beauty than students who did not receive such instruction. Individuals with a positive body image tend to filter and reject unrealistic images in the media to protect their body image (91).

The current study has several limitations worth noting. First, the age distribution of our sample was 18–34 years and is thus not representative of the general population; however, body image concerns are a key issue for this population, as eating disorders and associated pathology are common in this age group. In addition, the study design was cross-sectional; therefore, no longitudinal conclusions can be drawn about the relationships between these variables. These variables should be examined in the future using a prospective design to monitor sociocultural attitudes toward appearance and assess perceptions of eating attitude and negative appearance evaluation over time, particularly during the transition from adolescence to middle age. Despite these limitations, the current study provides useful insight into the relationships between eating attitudes, perceptions of one’s own body shape, and the factors associated with this social context in the Chinese population. We found that differing perceptions of external body shape can be effective predictors of eating behavior and social perception. Overall, studies on diet and related body image factors (internalized and perceived body acceptance) should be continued to improve the health of Chinese populations and combat eating disorders and weight-related diseases.

## Conclusion

Our results suggest that negative appearance ratings fully mediate the effect between dietary attitudes and sociocultural attitudes toward appearance. An individual’s eating attitudes influence negative body perceptions, which in turn affects the perception of their body shape. Our findings on the specific effects of dietary attitudes on body perception provide a psychological basis to guide and develop interventions on attitudes toward the body. Given the current cultural climate in which women’s and men’s bodies are constantly being objectified and censored, and recognizing the social importance of media (e.g., magazines and advertisements) in determining the ideals of beauty, there is a crucial need to identify the factors that influence individuals’ sociocultural attitudes toward appearance. In addition, studies using diverse age groups may help understand how these issues and behaviors manifest.

## Data Availability Statement

The original contributions presented in the study are included in the article/supplementary material, further inquiries can be directed to the corresponding authors.

## Ethics Statement

The studies involving human participants were reviewed and approved by Ethics Board for Research Projects at the Institute of Psychology, University of Gdańsk, Poland. The patients/participants provided their written informed consent to participate in this study.

## Author Contributions

HF, ML, MLa, BI, and YY: conceptualization. RW, ML, MLa, and YG: methodology. HF, JL, ML, MLa, BI, and SG: investigation. RW, YG, XW, and HF: writing – original draft preparation. RW, MLa, ML, BI, YG, XW, and HF: writing – review and editing. ML and MLa: supervision. ML: project administration. All authors contributed to manuscript revision, read, and approved the submitted version.

## Conflict of Interest

The authors declare that the research was conducted in the absence of any commercial or financial relationships that could be construed as a potential conflict of interest.

## Publisher’s Note

All claims expressed in this article are solely those of the authors and do not necessarily represent those of their affiliated organizations, or those of the publisher, the editors and the reviewers. Any product that may be evaluated in this article, or claim that may be made by its manufacturer, is not guaranteed or endorsed by the publisher.

## Footnotes

1
https://clinicaltrials.gov/ct2/show/NCT04432038
